# Effectiveness of Saline Nasal Irrigation in the Treatment of Rhinosinusitis and Allergic Rhinitis in Pediatric Patients: A Systematic Review

**DOI:** 10.7759/cureus.116769

**Published:** 2026-09-23

**Authors:** Mariana Salgado, Inês Rodrigues, Mariana Megre, Tiago Fernandes, Filipa Schedel, Rute Pereira, Catarina Correia, Catarina Machado, Catarina Alves, Tania Monteiro

**Affiliations:** 1 Family Medicine, Unidade Local de Saúde de Amadora/Sintra, Lisbon, PRT

**Keywords:** acute sinusitis, allergic rhinitis, child, nasal irrigation, pediatrics, quality of life, rhinosinusitis, saline solution, systematic review

## Abstract

Rhinosinusitis and allergic rhinitis are among the most common conditions of the upper airway in childhood, both presenting with nasal obstruction and rhinorrhea that can substantially impair quality of life. Alongside pharmacologic treatment, saline nasal irrigation has emerged as an inexpensive, accessible, and apparently well-tolerated nonpharmacologic measure, although its role in children remains incompletely defined. This systematic review aimed to evaluate the effectiveness of saline nasal irrigation in the treatment of rhinosinusitis and allergic rhinitis in the pediatric population. A systematic review was conducted in accordance with the PRISMA 2020 guidance. PubMed and the Cochrane Library were searched for randomized controlled trials and observational studies of saline nasal irrigation in children up to 18 years of age with rhinosinusitis or allergic rhinitis, compared with other effective medical therapy, published between 2014 and 2024 in English, Spanish, or Portuguese. Records were screened against predefined eligibility criteria, and data on study design, population, intervention, comparator, and outcomes were extracted. Of 50 records identified, 27 were removed for not meeting the PICO (population, intervention, comparator, and outcome) framework or for duplication, leaving 23 reports for full-text assessment; 21 were subsequently excluded, and two studies met all eligibility criteria. Both were prospective randomized controlled trials. The trial in acute sinusitis reported statistically significant between-group benefits favoring saline irrigation added to standard care, whereas the trial in allergic rhinitis and asthma reported significant within-group improvements in the irrigation group for several outcomes, without consistently significant between-group differences. In 60 children aged 3 to 12 years with acute sinusitis, all of whom completed the study, daily irrigation for three weeks significantly improved the nasal peak expiratory flow rate and pediatric rhinoconjunctivitis quality of life (p < 0.05), without a significant change in radiographic findings. In 20 children aged 6 to 18 years with allergic rhinitis and asthma, 12 weeks of twice-daily irrigation was associated with within-group improvements in bronchial hyperresponsiveness (PC20; p = 0.017), asthma control (p = 0.007), and disease-specific quality of life (p = 0.028) in the irrigation group, and reduced the proportion of moderate-to-severe persistent allergic rhinitis from 60% to 30% in that group (p = 0.003); the between-group difference in PC20 change was not statistically significant (p = 0.186), and forced expiratory volume in one second and fractional exhaled nitric oxide did not change significantly in either group. Reported adverse effects were minor and self-limited, including otalgia, aural fullness, and epistaxis, and did not compromise adherence. The available evidence indicates that adding saline nasal irrigation to standard treatment produces clinical and quality-of-life benefits in children with sinonasal disease, with a favorable safety profile. Given the small number of eligible studies and their methodological limitations, larger standardized trials are needed to consolidate these findings.

## Introduction and background

Allergic rhinitis and rhinosinusitis are among the most common conditions of the upper airway in childhood [1,2]. Both disorders share a symptomatic core of nasal obstruction and rhinorrhea and frequently coexist with, or contribute to, other respiratory conditions such as asthma and otitis media with effusion. Allergic rhinitis reflects an immunoglobulin E-mediated inflammatory response of the nasal mucosa to inhaled allergens [1], whereas rhinosinusitis is defined by inflammation of the nasal and paranasal sinus mucosa, presenting with two or more symptoms including nasal obstruction or discharge, with or without facial pain/pressure or reduction in smell; based on symptom duration, it is classified as acute (less than 12 weeks, with complete resolution) or chronic (12 weeks or longer) [2]. Although these conditions are rarely dangerous, they impose a considerable burden on affected children and their families.

The impact of pediatric sinonasal disease extends well beyond the nose. Persistent obstruction and rhinorrhea disturb sleep, impair concentration and school performance, and reduce overall quality of life; in children with comorbid asthma, poorly controlled upper-airway inflammation is associated with worse lower-airway control [1]. The chronicity of symptoms, the frequency of medical consultations, and the recurrent use of medication together make effective and sustainable management a priority in primary care and pediatric practice.

Standard management combines allergen avoidance, where feasible, with pharmacologic therapy. Intranasal corticosteroids, oral and intranasal antihistamines, leukotriene receptor antagonists, and, in the setting of bacterial rhinosinusitis, antibiotics form the mainstay of treatment [1,2]. These agents are effective but are constrained by difficulties with adherence, cost, potential adverse effects, and, in the case of antibiotics, legitimate concerns regarding overuse and antimicrobial resistance. There is therefore sustained interest in adjunctive nonpharmacologic measures that are safe, inexpensive, and easy to deliver at home.

Saline nasal irrigation consists of delivering an isotonic or hypertonic salt solution into the nasal cavity to rinse the mucosal surface. Proposed mechanisms of benefit include the mechanical clearance of mucus, allergens, and inflammatory mediators, improvement of mucociliary function, thinning of secretions, and reduction of mucosal edema, all of which may relieve obstruction and enhance the delivery and effect of topical medication [3,4]. The intervention is widely used, is generally regarded as well tolerated, and can be performed by caregivers after brief instruction [3,4].

Despite this widespread use, the evidence supporting saline nasal irrigation in children is less robust than in adults, in whom it is an established component of the management of sinonasal disease [3,4]. Pediatric studies are fewer, smaller, and heterogeneous with respect to the type of solution, the delivery device, the volume, and the treatment schedule, and although a meta-analysis in children with allergic rhinitis has suggested symptomatic benefit, the specific contribution of irrigation to outcomes in rhinosinusitis and allergic rhinitis has not been clearly established [5]. A focused synthesis of the pediatric evidence is therefore warranted. The present systematic review was undertaken to appraise and summarize the available controlled evidence on the effectiveness and tolerability of saline nasal irrigation in the treatment of rhinosinusitis and allergic rhinitis in children.

## Review

Methods

Study Design and Research Question

This review was designed and reported in accordance with the Preferred Reporting Items for Systematic Reviews and Meta-Analyses (PRISMA) 2020 statement [6]. The guiding question was whether the addition of saline nasal irrigation to standard care improves clinical and quality-of-life outcomes in children with rhinosinusitis or allergic rhinitis, compared with other effective medical therapy.

Eligibility Criteria

Eligibility was defined using the PICO (population, intervention, comparator, and outcome) framework. The population of interest was children up to 18 years of age with a diagnosis of rhinosinusitis or allergic rhinitis. The intervention was saline nasal irrigation using isotonic, hypotonic, or hypertonic solutions, delivered alone or as an adjunct to standard treatment, without a pre-specified irrigation technique, device, volume, or frequency. The comparator was other effective medical therapy, including standard pharmacologic treatment with or without placebo. The outcomes of interest were clinical benefit and tolerability, encompassing symptom severity, objective measures of nasal and lower-airway function, quality of life, medication use, and adverse effects. Eligible study designs were randomized controlled trials and observational studies.

Reports were excluded when they did not satisfy the PICO framework or were duplicates. On full-text assessment, further exclusions were applied to studies that evaluated irrigation combined with a pharmacologic agent rather than saline alone, examined conditions or populations outside the scope of the review, were conducted in adults, addressed perioperative irrigation, or represented secondary analyses such as systematic reviews and meta-analyses.

Search Strategy and Information Sources

Electronic searches were performed in PubMed (MEDLINE) and the Cochrane Library to identify randomized controlled trials and observational studies evaluating the benefits and tolerability of saline nasal irrigation in children with rhinosinusitis or allergic rhinitis. These two databases were selected as primary sources of controlled pediatric trials in this field and to minimize duplication of records across databases. The search was conducted in December 2024 combining the terms 'children' and 'nasal saline irrigation'; in PubMed, this was implemented as the MeSH-based search string (children[MeSH Terms]) AND (nasal saline irrigation[MeSH Terms]), retrieving 38 records, and the equivalent term combination was applied in the Cochrane Library, retrieving 12 records. Records were eligible when published between 2014 and 2024 and written in English, Spanish, or Portuguese, as these were the languages in which the review team could critically appraise full texts directly, avoiding translation-related inaccuracies.

Study Selection and Data Extraction

Records retrieved from the searches were screened by the review authors against the eligibility criteria. Titles and abstracts were first assessed through critical reading, and potentially relevant reports were then evaluated in full against the inclusion criteria. Screening of the 50 identified records and extraction of data from the included studies were performed independently by two reviewers, with disagreements resolved by a third reviewer. From each included study, data were extracted on the author and year, country, study design, sample size, participant age range, intervention and comparator, treatment schedule and irrigation method, outcomes assessed, and principal findings.

Risk of Bias

Risk of bias in the included randomized controlled trials was assessed using the Cochrane Risk of Bias tool for randomized trials (RoB 2), evaluating the domains of randomization process, deviations from intended interventions, missing outcome data, and measurement of the outcome. Each domain was rated as low risk, some concerns, or high risk, and an overall risk-of-bias judgment was derived for each study. 

Results

Study Selection

The database search identified 50 studies (Figure 1). After the removal of 27 records that did not satisfy the PICO framework or were duplicates, 23 articles were assessed in full text. A further 21 articles were excluded because they did not meet the inclusion criteria, most commonly because they evaluated irrigation combined with a pharmacologic agent rather than saline alone, addressed populations or conditions outside the scope of the review, were conducted in adults, concerned perioperative irrigation, or were secondary analyses. Two studies satisfied all eligibility criteria and were included in the qualitative synthesis [7,8]. Given the small number of eligible studies, the findings of this review should be interpreted as preliminary.

**Figure 1:**
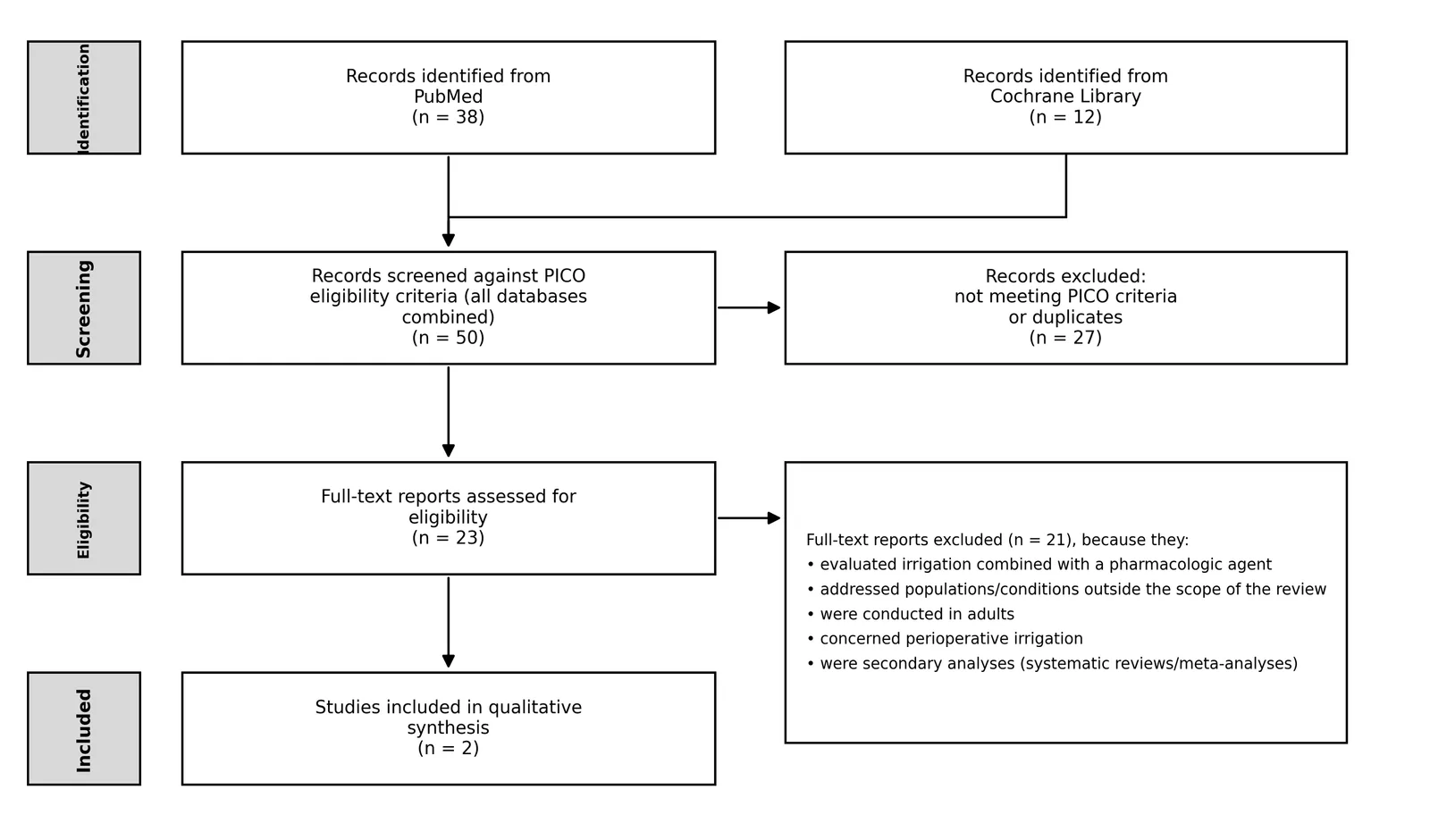
PRISMA 2020 flow diagram for the study selection process. PICO: Population, Intervention, Comparator, and Outcome; PRISMA: Preferred Reporting Items for Systematic Reviews and Meta-Analyses

Characteristics of the Included Studies

Both included studies were prospective, randomized, controlled trials that compared saline nasal irrigation added to standard therapy with standard therapy alone (Table 1) [7,8]. The first study enrolled 60 children aged 3 to 12 years with acute sinusitis [7]. Participants were randomized to normal-saline nasal irrigation, performed one to three times daily for three weeks in addition to standard treatment comprising systemic antibiotics, mucolytics, and nasal decongestants, or to standard treatment without irrigation [7]. Outcome assessment included a nasal peak expiratory flow rate test, nasal smear examination, sinus radiography, a daily symptom diary, and the Pediatric Rhinoconjunctivitis Quality of Life Questionnaire, obtained at baseline and repeated over weekly follow-up visits [7]. The second study enrolled 20 children aged 6 to 18 years with allergic rhinitis and comorbid asthma [8]. The intervention group performed saline nasal irrigation twice daily for 12 weeks in addition to pharmacologic therapy comprising levocetirizine, an inhaled corticosteroid, and, in some children, montelukast, while the control group received the same pharmacologic therapy without irrigation [8]. Outcomes measured at baseline and at 12 weeks included the forced expiratory volume in one second, the methacholine provocation concentration causing a 20% fall in this value (PC20), fractional exhaled nitric oxide, the Asthma Control Test, allergic rhinitis severity classified according to the Allergic Rhinitis and its Impact on Asthma criteria, a disease-specific pediatric quality-of-life questionnaire, and the proportion of eosinophils in nasal secretions [8].

**Table 1:** Characteristics of included studies. RCT: randomized controlled trial; FEV1: forced expiratory volume in one second; PC20: provocation concentration causing a 20% fall in FEV1; FeNO: fractional exhaled nitric oxide; ARIA: Allergic Rhinitis and its Impact on Asthma; QoL: quality of life.

Study	Design/population	Sample size	Age range	Outcomes measured
Wang et al., 2014 [7] Taiwan	Prospective RCT. Acute sinusitis in atopic children; irrigation added to standard treatment (antibiotics, mucolytics, decongestants) vs. standard treatment alone.	n = 60 randomized n = 60 completed protocol	3–12 years	Nasal peak expiratory flow rate, nasal smear examination, sinus radiography (Water's projection), daily symptom diary (total symptom score), pediatric rhinoconjunctivitis Quality of Life Questionnaire
Jung et al., 2020 [8] South Korea	Prospective RCT. Allergic rhinitis with comorbid asthma; irrigation added to pharmacologic therapy (levocetirizine, inhaled corticosteroid, ± montelukast) vs. pharmacologic therapy alone.	n = 20 randomized	6–18 years	FEV1 PC20 (methacholine provocation), fractional exhaled nitric oxide (FeNO), asthma control test, ARIA severity classification, disease-specific pediatric QoL questionnaire, nasal eosinophil proportion

Risk of Bias in Included Studies 

Both included trials were judged to be at overall high risk of bias (Table 2). The principal source of risk in both studies was the absence of blinding of participants, caregivers, and outcome assessors, which may have influenced subjectively reported outcomes such as symptom diaries and quality-of-life questionnaires. Missing outcome data were low risk in both included trials, as no participants were lost to follow-up in either the study by Wang et al. [7] or that by Jung et al. [8].

**Table 2:** Risk-of-bias assessment (Cochrane RoB 2). D1–D4 correspond to the core Cochrane RoB 2 domains for individually randomized, parallel-group trials. Both trials were rated overall 'High risk' primarily because participants and caregivers could not be blinded to allocation (open-label irrigation), which may have influenced subjectively assessed outcomes (e.g., symptom diaries, quality-of-life questionnaires). 'Missing outcome data' was rated 'Low' for both trials, as no participants were lost to follow-up in the study by either Wang et al. or Jung et al. 'Measurement of outcome' was rated 'Some concerns' for the study by Wang et al., reflecting blinded assessment of radiographic and nasal smear findings alongside unblinded, self-reported symptom and quality-of-life measures, and 'High' for the study by Jung et al., in which outcome assessment relied more broadly on unblinded, patient-reported measures.

Study	D1: Randomization process	D2: Deviations from intended intervention	D3: Missing outcome data	D4: Measurement of outcome	Overall
Wang et al., 2014 [7]	Some concerns	High	Low	Some concerns	High
Jung et al., 2020 [8]	Some concerns	High	Low	High	High

Synthesis of Results 

The trial of acute sinusitis demonstrated statistically significant between-group benefits favoring the addition of saline nasal irrigation, whereas the trial of allergic rhinitis and asthma demonstrated significant within-group improvements in the irrigation group, without consistently significant between-group differences [7,8]. In children with acute sinusitis, the irrigation group showed significant improvements in the nasal peak expiratory flow rate and in PRQLQ scores compared with the control group (p < 0.05 for both), with significantly greater improvement in nasal congestion and sleep quality, although most other individual symptom scores did not differ significantly between groups; radiographic findings did not differ significantly between the groups (p > 0.05) [7]. In children with allergic rhinitis and asthma, 12 weeks of saline nasal irrigation was associated with a significant within-group improvement in bronchial hyperresponsiveness, reflected by a higher PC20 at week 12 than at baseline in the irrigation group (p = 0.017) but not in the control group (p = 0.333); the median change in PC20 did not differ significantly between the groups (p = 0.186) [8]. The Asthma Control Test score improved after 12 weeks of irrigation (p = 0.007), and the disease-specific quality-of-life score improved relative to baseline in the irrigation group (p = 0.028) [8]. The proportion of children with moderate-to-severe persistent allergic rhinitis fell from 60% to 30% in the irrigation group (p = 0.003), with no significant change in the control group (p = 0.094) [8]. The forced expiratory volume in one second, fractional exhaled nitric oxide, and the proportion of eosinophils in nasal secretions did not change significantly in either group (all p > 0.05) [8].

Adverse effects reported across the included studies were minor and self-limited, including otalgia, aural fullness, and epistaxis, and did not compromise treatment adherence [8]. No significant adverse effects were reported in the acute sinusitis trial [7]. In the trial of children with allergic rhinitis and asthma, no asthma exacerbations occurred, and three children in the irrigation group reported otalgia, a sensation of aural fullness, or epistaxis, each of which resolved within three days without the need for medication [8].

Discussion

This review identified two small randomized controlled trials of saline nasal irrigation added to standard care in children [7,8]. In acute sinusitis, irrigation was associated with significant between-group improvements in nasal airflow and rhinoconjunctivitis-related quality of life. In children with allergic rhinitis and asthma, irrigation was associated with significant within-group improvements in bronchial hyperresponsiveness, asthma control, and allergic rhinitis-related quality of life, without consistently significant between-group differences. The magnitude and pattern of benefit thus varied with the clinical context, the outcomes assessed, and the type of comparison performed.

These findings are consistent with adult evidence, in which irrigation is an established, inexpensive adjunct for rhinosinusitis and allergic rhinitis [3,4], and they are biologically plausible: irrigation clears mucus, allergens, and inflammatory mediators, reduces mucosal edema, and supports mucociliary clearance. Because it is inexpensive, easy to perform at home, and associated only with minor, self-limited adverse effects [8], irrigation is an attractive addition to routine care, with the potential to reduce reliance on medication.

Several caveats apply to these findings, discussed in detail below. Adequately powered, standardized pediatric trials with validated outcomes and longer follow-up are needed to confirm these findings and to define the optimal solution, volume, device, and frequency. 

Strengths and Limitations

The principal strength of this review is its focused clinical question and its adherence to PRISMA 2020 guidance, with a transparent screening process applied to a defined pediatric population [6]. By restricting eligibility to controlled studies of saline irrigation and by extracting objective as well as patient-reported outcomes, the review provides a clear appraisal of the most directly relevant evidence.

The review also has important limitations, the foremost of which is the very small number of eligible studies. Only two trials met the inclusion criteria, both with small samples and an absence of blinding. Wang et al. did not report treatment adherence rates or stratify results by disease severity, and Jung et al. did not account for seasonal variation in allergic rhinitis symptoms, a potential confounder given the 12-week follow-up period. The two trials also addressed different conditions, which prevented meta-analysis and limits the generalizability of the findings. In addition, the two included trials differed in irrigation technique, device, volume, and frequency, both used isotonic saline, though the eligibility criteria did not restrict tonicity, which limits direct comparability between studies and precludes recommendations regarding an optimal irrigation regimen.

This review was not registered prospectively in a public database such as PROSPERO, which is a limitation with respect to current best-practice recommendations for systematic reviews. In addition, this review did not include a formal assessment of the overall certainty of evidence (e.g., using the GRADE approach), which was beyond the scope of the risk-of-bias evaluation performed and was further limited by the small number and heterogeneity of the included studies. The search was restricted to two databases and to studies with extractable outcomes in the conditions of interest, so relevant evidence may have been missed. Additionally, restricting eligible records to English, Spanish, or Portuguese may have excluded relevant studies published in other languages, including in Asian populations where pediatric rhinosinusitis and allergic rhinitis research is active.

## Conclusions

This systematic review found that both included trials were at high risk of bias, most notably due to the absence of blinding. Within this limited evidence base, saline nasal irrigation added to standard therapy was associated with clinical and quality-of-life improvements in children with sinonasal disease, including in nasal airflow, rhinoconjunctivitis-related quality of life, bronchial hyperresponsiveness, and asthma control across the two eligible trials. The intervention was inexpensive, easy to administer at home, and associated only with minor and self-limited adverse effects that did not compromise adherence. Saline nasal irrigation may therefore be considered a reasonable and low-risk adjunct to conventional pharmacologic treatment in children with rhinosinusitis and allergic rhinitis, although the certainty of this evidence remains limited. 

These conclusions should be interpreted with the caution warranted by the limitations discussed above, including the heterogeneity in irrigation technique, device, volume, and frequency between the two included trials. Larger, methodologically rigorous, and standardized pediatric trials are required to confirm the effectiveness of saline nasal irrigation, to define the optimal solution and delivery regimen, and to clarify its specific role in rhinosinusitis. Until such data are available, clinicians can reasonably offer saline nasal irrigation as a safe supportive measure while individualizing treatment, since no single irrigation technique has yet been established as superior in children.
